# How perceptions of labor market opportunities predict happiness: evidence from natural field experiments

**DOI:** 10.3389/fsoc.2025.1527125

**Published:** 2025-04-24

**Authors:** Roger Fernandez-Urbano

**Affiliations:** European University Institute (EUI), Fiesole, Italy

**Keywords:** happiness, perceptions, labor market, culture, natural field experiments, social background, subjective wellbeing

## Abstract

**Introduction:**

Subjective variables related to the labor market have long been recognized to be strongly associated with individuals' happiness. However, most existing research relied on correlational analyses, which have been insufficient in establishing causation. Establishing causal links is crucial for disentangling reverse causality as well as addressing order-of-effect and omitted variable biases, thereby determining whether and how subjective labor market perceptions directly impact happiness. Moreover, prior studies have primarily focused on personal labor market concerns, largely overlooking perceptions of labor market opportunities at the macro level. Recognizing these broader perceptions is vital for understanding behavioral outcomes at both individual and societal levels, and for understanding persistent levels of structural unemployment and particular policy preferences.

**Methods:**

This study addresses these gaps by conducting harmonized natural field experiments in Pennsylvania, U.S., and Barcelona, Spain, to examine how perceptions of macro-level labor market opportunities impact happiness.

**Results and discussion:**

The results reveal that in Barcelona only positive perceptions have an effect and increase happiness, whereas in Pennsylvania only negative perceptions impact happiness and do so detrimentally. This discrepancy is attributed to subtle, experimentally induced shifts in the framing of truthful macroeconomic information. The discussion proposes a cultural bias mechanism to account for these differences. Heterogeneous effects of the treatments in terms of social background and subjective health are also discussed. The findings hold significant implications for policymaking and organizational strategies, underscoring the importance of understanding how perceived macro-labor market opportunities shape happiness.

## 1 Introduction

Subjective variables related to the labor market, such as labor market insecurity, job satisfaction or individual unemployment, have long been recognized to be strongly associated with individuals' happiness (Clark et al., 2018; Dolan et al., 2008; Jahoda, 1982). However, the existing literature reveals two critical gaps. Firstly, establishing causality remains a significant challenge. Much of the existing literature relies on observational studies using regression models applied to survey data, which, despite controlling for various factors, remain limited in their ability to establish causality (Fernandez-Urbano, 2024). In particular, these studies are vulnerable to several biases. One of the most important concerns is reverse causality, a persitent challenge in happiness research (Layard and De Neve, 2023; Cannas et al., 2019). Intrinsically happy individuals might report a more optimistic outlook on economic circumstances, making it difficult to determine whether positive subjective labor market variables lead to increased happiness or whether higher wellbeing influences these perceptions (Dolan et al., 2008; Forgeard et al., 2011). As Krause (2013) argues, subjective wellbeing is highly susceptible to reverse causality, particularly in research involving employment-related variables. Similarly, using instrumental variable analysis, Rode and Coll (2012) found that while economic freedom appear to influence subjective wellbeing, a long-term effect of populations' subjective wellbeing on preferences for economic freedom cannot be rulet out. This issue is closely related to order-of-effects bias in correlational studies, which do not allow for a clear establishment of the temporal sequence of cause and effect (Jackson and Cox, 2013). Additionally, omitted variable bias poses a serious limitation. Unobserved factors—such as personality traits, innate optimism, or social support—may simultaneously influence subjective labor market variables and happiness, confounding the observed relationships (Ferrer-i-Carbonell, 2013; Ferrer-i-Carbonell and Frijters, 2004).

Given these challenges, establishing causal relationships is crucial for determining whether and how subjective labor market perceptions directly impact happiness. This is is not only essential for advancing theoretical clarity but also for informing effective policy. Without clear evidence on causal pathways, interventions may be misdirected or ineffective. Identifying which variables merely correlate with happiness and those that exert a causal effect is essential for disentangling the determinants of wellbeing (OECD, 2013) and for understanding how exactly social and economic contexts shape individuals' lives (Dolan and White, 2007). In the light of this, this, it is important to note that happiness and subjective wellbeing share an almost identical conceptual structures (Blanchflower and Oswald, 2004; Diener et al., 2006), which is why this study use both terms interchangeably.

Secondly, most studies have primarily focused on labor market variables that capture individuals' concerns regarding their personal work experiences. These include individual job insecurity (Burchell, 2011; Carr and Chung, 2014; Geishecker, 2012; Knabe and Rätzel, 2011), individual socioeconomic circumstances (Brown et al., 2005; Johnson and Krueger, 2006; Otis, 2017), individual economic uncertainty (Campara et al., 2017; Tonzer, 2019) or employability (Gowan, 2012; Green, 2011). This literature has contributed by showing that there is a link between subjective wellbeing and the perceptions that individuals have, but primarily on perceptions of individually experienced labor market consequences, such as ‘losing the current job'. However, there has been limited exploration of individuals' perceptions of labor market opportunities at the macro level, which refers to individuals' perceptions of overall available labor market opportunities in their region or country. Investigating these broader perceptions is important for understanding the behavioral consequences that can generate both at the individual and societal level and can shed light on understanding particular policy preferences and persistent levels of structural unemployment in societies (Graham and Pettinato, 2004). For instance, individuals who have positive perceptions of such opportunities may follow their desired career path more quickly in or outside their actual labor sector, as well as pursuing other personal goals, like parenthood or changing residence (Forgeard et al., 2011; Vignoli et al., 2020). In contrast, individuals who have negative perceptions may experience persistent levels of stress and uncertainty, making them conservative (Stiglitz et al., 2018), prompting them to stay in jobs they dislike (Guzzo and Hayford, 2020; Mills et al., 2011) and to consider migrating to other countries (Hendriks and Bartram, 2016; Hendriks, 2015). Similar dynamics can occur at the societal level. While positive perceptions may advance social integration and stability (Dolan et al., 2008; Dolan and White, 2007), negative ones can increase support for populist parties, hinder recovery after macroeconomic recessions, and discourage entrepreneurship (Stiglitz et al., 2018; Giugni and Grasso, 2018).

To address these significant gaps in the existing literature, this study employs two harmonized natural field experiments to explore how perceptions of macro-level labor market opportunities impact happiness. The experiments were conducted in two WEIRD (Western, Educated, Industrialized, Rich, and Democratic) contexts (Henrich et al., 2010) with distinct historical trajectories of structural unemployment and labor market regulations: East Stroudsburg, Pennsylvania (U.S.), and Barcelona, Catalonia (Spain). Experimental designs are particularly effective for establishing causal relationships by mitigating the influence of unobserved variables (Jackson and Cox, 2013). The study also examines heterogeneous effects of the treatments in terms of gender, social background and subjective health (see Section 3.2).

To ensure robustness, the experiments were conducted during periods of relative macroeconomic stability—approximately a decade after the 2008 economic crisis and before the COVID-19 pandemic (late 2018 to early 2019). During these periods, key macroeconomic indicators such as unemployment rates and GDP growth were relatively stable in both regions. For a more detailed justification and statistical context for both regions, please refer to Supplementary File A. The experiments were embedded in the context of university exam sessions, where the framing of the last exam question was subtly manipulated to provide truthful but differently framed information (positive, negative, or neutral) on macro-level labor market opportunities. Subsequently, students were asked to anonymously complete a routine questionnaire addressing their wellbeing and mental health. This approach enabled a controlled examination of the effects of labor market perceptions on happiness, ensuring high internal validity to effectively capture potential causal pathways. The trade-off between internal and external validity is further discussed in the limitations section. Additional details on the methodology and contextual variables are provided in the next section and in the Supplementary material.

Information provision experiments are increasingly used in social sciences to examine how objective information about socioeconomic realities contrasts with individuals' prior perceptions and influences their cognitive states, judgements, and behaviors (Haaland et al., 2023). Previous findings indicate that these exogenous information treatments–framed either positively or negatively– can generate substantial shifts in attitudes and preferences (e.g., see Kuziemko et al., 2015; Cavallo et al., 2016; Roth and Wohlfart, 2020). In the context of macro-labor market perceptions, such shifts may impact subjective wellbeing, given that previous correlational analyses consistently link subjective wellbeing and variables related to perceived macro-labor market opportunities (e.g., see Layard and De Neve, 2023; Lepinteur, 2024). Therefore, it could be expected that exposure to positively framed information about macro–labor market opportunities may have a positive impact on happiness, whereas exposure to negatively framed information could result in lower happiness levels relative to the neutral condition.

## 2 Materials and methods

Two harmonized natural field experiments involved a randomized assignment of ‘perceiving fewer or more macro-labor market opportunities' across both study contexts. Consistent with established cross-country experimental methodologies (e.g., Brandts et al., 2004; Gërxhani and Schram, 2006; Gërxhani et al., 2022), this research employed rigorous experimental controls to mitigate between-country disparities stemming from external constraints. The experimental cohorts comprised native 3rd- and 4th-year undergraduates specializing in social sciences disciplines, encompassing fields such as business administration, political science, international relations, economics, sociology, or double degrees, drawn from the University of Barcelona and East Stroudsburg University of Pennsylvania. Notably, the experiments were conducted within the students' natural environment, coinciding with official examination sessions. This methodological approach was adopted to circumvent the primary limitations associated with traditional laboratory or lab-in-the-field experiments, namely, the observer effect and self-selection biases among participants, as well as the constraints associated with natural field experiments, such as potential communication between subjects during the experimental process (see Barrera et al., 2024).

As detailed in Section 2.4 (Measures), the core of the experimental design revolves around an information treatment introduced in the final question of an exam. This treatment was designed to experimentally generate exogenous variation in students' perceptions of macro-labor market opportunities by providing them with truthful information about these opportunities. Following this, students completed a routine subjective wellbeing questionnaire, which both universities occasionally administer. Information provision experiments are particularly valuable as they enable researchers to induce exogenous variation in perceptions of real-world phenomena (Haaland et al., 2023).

The information treatment in this study draws inspiration from the work of Wiswall and Zafar (2015), who examined causal relationships between income expectations and educational choices. Wiswall and Zafar studied the determinants of college major selection using an experimentally generated panel of beliefs. These beliefs were shaped by providing students with accurate information about the true population distribution of various major-specific characteristics. For instance, participants in their study completed a survey in which one treatment group received factual information on population wage outcomes associated with different educational paths. This intervention was designed to shift perceptions about future income linked to potential educational decisions (e.g., “Did you know that if you study biology, you are expected to earn X€ in the labor market?”).

Although information provision experiments remain relatively uncommon, they have gained prominence over the past decade, especially in several sub-fields of economics (Haaland et al., 2023). Examples include experimental designs evaluating perceptions of macroeconomic factors such as income inequality (Kuziemko et al., 2015), inflation expectations (Cavallo et al., 2016), or recession expectations (Roth and Wohlfart, 2020). They have also been applied to more microeconomic perceptions, such as consumer valuations of nutritional information when shopping (Gracia et al., 2009) or voting behavior (Gerber et al., 2020).

To the best of my knowledge, this study represents the first cross-country information provision natural field experiment in the subjective wellbeing literature within the social sciences.

The selection of the University of Barcelona and East Stroudsburg University of Pennsylvania as research sites was deliberate, motivated by several key factors. Firstly, both institutions are esteemed public universities renowned within their respective regions, with a long-standing tradition of admitting students from diverse socioeconomic backgrounds. However, to ensure that all participants shared local social and cultural experiences, we focused on courses exclusively attended by native students, as verified through the universities' enrolment records. Consequently, no international students were enrolled in these specific classes, and thus none were excluded from the final sample. This aspect helps mitigate concerns regarding geographic representativeness in the study, as all participants are drawn from the regions where each university is located.

While universities—particularly public institutions—tend to facilitate broader access to higher education, this study acknowledges that attending university still remains a relatively privileged experience. Nonetheless, by selecting public universities rather than private, highly selective institutions, this study aims to minimize issues of sample homogeneity, particularly the overrepresentation of students from higher socioeconomic strata. Additionally, it is important to clarify that East Stroudsburg University of Pennsylvania is distinct from the University of Pennsylvania (Penn). While the latter is a private Ivy League institution based in Philadelphia, East Stroudsburg University is a public university within the Pennsylvania State System of Higher Education, emphasizing accessibility and regional diversity in student admissions. More information regarding implementing partners can be found in Supplementary File A, while additional descriptive analyses are provided in Supplementary File C.

Furthermore, the comparability of grading systems across both universities played a pivotal role in their selection. Notably, undergraduate programs in the social sciences at both institutions adhere to a similar grading structure. Students typically undergo assessment through a series of partial exams throughout each course, culminating in a final examination. The final grade is often determined based on the aggregation of the highest scores attained in these partial exams, which typically contribute to 50 or 60% of the overall grade. This grading system fosters intrinsic motivation among students to excel in each partial exam, which predominantly consists of multiple-choice or open-question formats assessing concepts covered in preceding lectures.

### 2.1 Experimental manipulation

The experimental manipulation was seamlessly integrated into one of the partial exams administered to students. This exam, consistent across both universities, consisted of five multiple-choice questions, as detailed in Supplementary File D. To mitigate any potential impact on students' performance in the remainder of the exam, the experimental treatment was strategically positioned as the final question. Specifically, this question aimed to influence participants' perceptions of macro-labor market opportunities. Students were randomly assigned to either the control group or one of two treatment groups (positive or negative). Further details are provided below. Following completion of the exam, immediately after the experimental question, all students were invited to anonymously respond to a questionnaire pertaining to their wellbeing. This methodological approach facilitated a comparative analysis of happiness levels between participants assigned to the treatment groups and those in the control group.

It is worth noting that both universities routinely administer anonymous questionnaires to assess student wellbeing and mental health. This institutional practice likely contributed to students perceiving the completion of this questionnaire as standard, reducing potential response biases and encouraging truthful reporting (see Section 2.4 “Measures” for a more detailed Discussion).

All participants were undergraduate students enrolled in modules pertinent to either the Catalan-Spanish-European (University of Barcelona) or U.S. labor markets (East Stroudsburg University). Given the thematic alignment of the experimental content with their coursework, participants were unlikely to harbor suspicions regarding the nature of the experimental question posed in the exam. Moreover, both universities granted the author permission to conduct a session on Spanish-European and U.S. labor markets 1 week preceding the exam, thereby ensuring his presence during the exam was perceived as natural. It is pertinent to note that both university departments frequently host guest speakers to deliver specialized lectures within their respective domains, thereby making the presence of a lecturer during exam sessions a normative occurrence.

#### 2.1.1 Sample

In the University of Barcelona sample, a total of 147 native individuals participated in the natural field experiment. Specifically, 49 individuals (34.01%) were randomly exposed to positive information, 49 individuals (34.01%) to negative information, and 49 individuals (34.01%) to neutral information (control group). In the East Stroudsburg University sample, the experiment involved 172 native individuals, with 63 (36.63%) randomly exposed to positive information, 52 (30.23%) to negative information, and 57 (33.14%) to neutral information. A detailed description and comparison of the socio-demographic characteristics of the sample—including social origin, subjective health, age, and gender—is available in Supplementary File C.

### 2.2 Transparency and openness

Participation numbers align with the power analysis conducted prior to the experiment, which determined the desired number of participants per cell (positive/negative/neutral) for each context (see Supplementary File B). Ethical approval for the experiments was diligently secured from the Bioethics Commission of the University of Barcelona and the Institutional Review Board of East Stroudsburg University of Pennsylvania. For the latter, it was required to successfully pass the Collaborative Institutional Training Initiative (CITI) online module called the “Human Subjects Research – Social-Behavioral-Educational Basic.” In addition, a pilot session involving doctoral and postdoctoral researchers from the Department of Political and Social Sciences at the European University Institute was conducted prior to the main experiment. Following the session, the author presented the experimental design, planned analytical procedures, and power analysis. The data and analysis code are available at article/Supplementary material. Data were analyzed using STATA, version 17.

### 2.3 Procedure

Upon entering the examination room, the author allocated participants to different parts of the room randomly, a common practice employed to deter plagiarism and an integral component of the students' exam routine. This randomization process served to minimize the likelihood of students with similar characteristics being exposed to the same treatment, such as friends from the same socioeconomic background, age group, or gender, who might have otherwise chosen to sit in close proximity to each other (see also Tables 1, 2 for balance tests on the pre-treatment characteristics of the participants). It is noteworthy that the random assignment of students to their seats occurred upon their physical entry into the examination room to maintain consistency with the standard examination protocol at both universities and preserve the natural setting of the field experiment. Participants were instructed to use a pen and maintain silence until the commencement of the exam.

**Table 1:** University of Barcelona (Spain) sample characteristics.

		**(1)**		**(2)**		**(3)**	***t*-test**	***t*-test**	***t*-test**
		**Negative I**		**Neutral I**		**Positive I**	***p*** **-value**	***p*** **-value**	***p*** **-value**
**Variable**	**N**	**Mean/SE**	**N**	**Mean/SE**	**N**	**Mean/SE**	**(1)-(2)**	**(1)-(3)**	**(2)-(3)**
Age	49	21.082	49	21.633	49	21.347	0.347	0.560	0.638
		[0.300]		[0.501]		[0.340]			
Gender	49	1.224	49	1.367	49	1.286	0.124	0.492	0.394
		[0.060]		[0.070]		[0.065]			
Social Background	49	2.776	49	2.939	49	2.816	0.358	0.821	0.498
		[0.125]		[0.125]		[0.129]			
Subj. Health	49	1.878	49	2.020	49	1.796	0.397	0.614	0.138
		[0.126]		[0.111]		[0.101]			

Robust standard in parentheses. The value displayed for *t*-tests are *p*-values.

**Table 2:** East Stroudsburg University (U.S.) sample characteristics.

		**(1)**		**(2)**		**(3)**	***t*-test**	***t*-test**	***t*-test**
		**Negative I**		**Neutral I**		**Positive I**	***p*** **-value**	***p*** **-value**	***p*** **-value**
**Variable**	**N**	**Mean/SE**	**N**	**Mean/SE**	**N**	**Mean/SE**	**(1)-(2)**	**(1)-(3)**	**(2)-(3)**
Age	52	21.000	57	20.140	63	20.048	0.254	0.184	0.813
		[0.717]		[0.292]		[0.262]			
Gender	52	0.481	57	0.421	63	0.429	0.536	0.580	0.934
		[0.070]		[0.066]		[0.063]			
Social Background	52	3.135	57	3.158	63	3.175	0.887	0.791	0.906
		[0.123]		[0.109]		[0.092]			
Subj. Health	52	1.769	57	1.702	63	1.937	0.619	0.227	0.093^*^
		[0.094]		[0.097]		[0.098]			

Robust standard in parentheses. The value displayed for *t*-tests are *p*-values. ^*^ indicates significance at the 10 percent level.

Five minutes prior to the exam's commencement and once all students were seated, the exam papers and the subjective wellbeing questionnaires—positioned physically beneath the exam papers—were distributed. Subsequently, students were informed that they had 30 min to complete the exam and that upon completion, they could spend up to 5 min answering the subjective wellbeing questionnaire. Additionally, students were advised that, to minimize disruption, they were not permitted to leave the room until all students had completed their exams.

Following the completion of both the exam and the questionnaire by all students, the two documents were collected and placed in separate containers. Subsequently, students were informed of their participation in an experiment, and their consent and written authorization were solicited. Notably, 98% of University of Barcelona students and 98% of East Stroudsburg University students completed both the exam and the subjective wellbeing questionnaire and provided consent (refer to Supplementary File D for comprehensive exam session instructions in both contexts). Finally, participants were assured that their exams would be evaluated for their module grades as usual. After addressing any questions pertaining to the experiment, participants were thanked and dismissed.

### 2.4 Measures

#### 2.4.1 Perceptions of macro labor market opportunities

Consistent with Wiswall and Zafar's (2015) approach, the experimental treatment manipulates the framing of information to which participants are exposed, encompassing three types: positive, negative, and neutral. The neutral framing serves as the control treatment and functions as the reference category in subsequent regression analyses. These treatments are rooted in truthful information, with variations solely in the applied framing. While the framing necessitates similar wording of the manipulation, it also requires sufficient adaptation to the specific contexts and languages (refer to Supplementary File A for comprehensive details). Overall, the wording and order of information remain consistent across specific contexts and treatments, with Supplementary File D providing a thorough description of variations between exam versions in both contexts.

##### 2.4.1.1 Treatment variable 1: positive information framing

Positive perceptions of macro-labor market opportunities are induced through a favorable framing of actual information on the labor market. In the University of Barcelona, this positive framing is articulated as follows: “Question 5. *Fortunately, employment rates in Spain have been firmly rising. Furthermore, after one decade, employment rates are expected to soon reach the same levels as in 2008*. At what moment did Spain begin to increase its private and public debt to invest in the real-estate market and start the process of deindustrialization that led to the state of the economy of 2008?” Answer categories include: *(a) At the end of the Franco Regime; (b) At the beginning of the 1980s; (c) At the beginning of the 2000s; (d) During 2006 and 2007*.

The slightly modified version tailored to East Stroudsburg University is presented as follows: “Question 5. *Fortunately, in the U.S. context, employment rates have been rising steadily and have already reached the same levels as in 2008. This fast recovery has been characterized by an increased tertiary sector and a decreased industrial sector*. In which moment did the U.S. experience the most important process of deindustrialization?” The answer categories are: (a) *During the 1960s; (b) During the 1980s; (c) During the 2000s; (d) During 2006 and 2007*.

##### 2.4.1.2 Treatment variable 2: negative information framing

Negative perceptions of macro-labor market opportunities are engendered through an adverse framing of actual labor market information. In the University of Barcelona, this negative framing is articulated as follows: “Question 5. *Unfortunately, unemployment rates in Spain are still among the highest in Europe. Furthermore, after one decade, unemployment rates have not yet reached the levels prior to the 2008 Economic Crisis*. When did Spain begin to increase its private and public debt to invest in the real-estate market and start the process of deindustrialization that lead to its 2008 Economic Crisis?” The answer categories include: *(a) At the end of the Franco Regime; (b) At the beginning of the 1980s; (c) At the beginning of the 2000s; (d) During the 2 previous years before the 2008 Economic Crisis*.

Similarly, the negative information framing for East Stroudsburg University is formulated as follows: “Question 5. *Unfortunately, in the U.S. context, unemployment also continues to be an important individual and macroeconomic problem. The recovery from the 2008 Economic Crisis has been very slow. Furthermore, during the last decades, the U.S. economy has been characterized by a relatively decreased industrial sector*. In which moment did the U.S. experience the most important process of deindustrialization?” The answer categories are: *(a) During the 1960s; (b) During the 1980s; (c) During the 2000s; (d) Only during the 2 previous years before the 2008 Economic Crisis*.^1^

##### 2.4.1.3 Treatment variable 3: Control treatment: neutral information

Students randomly assigned to the control treatment encounter a neutral framing of actual macro-labor market information. In the University of Barcelona this is: “Question 5*: At what moment did Spain begin to increase its private and public debt to invest in the real-estate market and start the process of deindustrialization that lead to the state of the economy of 2008?”* The answer categories mirror those in the positive framing treatment at the University of Barcelona.

The control treatment at East Stroudsburg University is introduced as follows: “Question 5. *In the U.S. context, during the last decades, the economy has been characterized by an increasing tertiary sector and a decreasing industrial sector. In which moment did the U.S. experience the most important process of deindustrialization?”* Here too, the answer categories mirror those in the positive framing treatment at East Stroudsburg University.

#### 2.4.2 Happiness

An anonymous survey consisting of 18 questions was administered to students (see Supplementary File E). The survey asked different wellbeing facets, following this order: happiness, engagement-flow, relationships, meaning and purpose in life, and accomplishment and competence. The measure of happiness in this study is rooted in the survey question regarding happiness: “Taking all things together, how happy would you say you are? Note that 0 is Extremely Unhappy and 10 is Extremely Happy,” with response options spanning from 0 to 10. This measure is widely used to capture global levels of subjective happiness (Layard and De Neve, 2023), allowing individuals to assess their overall happiness based on the aspects they personally consider most important (Clark et al., 2018). Although the question explicitly asks about *happiness*, which may imply an affective dimension, the literature predominantly classifies this type of measure as evaluative because it prompts individuals to make a broad cognitive judgment about their life circumstances (Kahneman and Krueger, 2006; OECD, 2013). The “taking all things together” framing encourages respondents to reflect on their overall wellbeing rather than reporting momentary affect. Thus, while happiness measures like this one may capture some hedonic elements, they are generally considered part of the evaluative wellbeing domain, distinct from short-term affective states (Diener et al., 2018).

The questionnaire was completed anonymously, and students were explicitly informed that their responses would remain confidential and have no bearing on their academic evaluations. Ensuring anonymity is an important factor in mitigating social desirability bias, as self-administered surveys reduce external pressures that may lead to misreporting on sensitive topics (Tourangeau and Yan, 2007). While self-reported wellbeing measures are not entirely free from bias, previous research has shown that student responses to wellbeing surveys are generally reliable and internally consistent, particularly when they are accustomed to completing such questionnaires (Lucas and Brent Donnellan, 2012). Additionally, similar single-item happiness measures have been extensively validated in large-scale surveys, such as the European Social Survey and prestigious reports such as the World Happiness Report, further supporting their credibility as meaningful indicators of subjective wellbeing.

Moreover, studies indicate that numerical wellbeing reports reliably correlate with individuals' psychological reactions, physiological markers, and observable behaviors, suggesting that they meaningfully reflect inner wellbeing (Pavot et al., 1991). For instance, individuals with higher self-reported happiness tend to smile more (Ekman et al., 1990) and exhibit higher electrical activity in the brain's prefrontal areas, which are responsible for processing emotional experiences (Sutton and Davidson, 1997). This body of research provides strong empirical support for the validity of self-reported happiness measures (Diener et al., 2018).

Although the possibility of differential reporting bias across subgroups within each of the study samples (University of Barcelona and East Stroudsburg University) cannot be ruled out, extensive research on self-reported measures of happiness has demostrated high reliability and consistency across diverse population groups (Kahneman and Krueger, 2006; Lucas and Brent Donnellan, 2012). Moreover, because participants were randomly assigned to experimental conditions, any systematic differences in response tendencies across subgroups are unlikely to bias the estimated treatment effects (Gërxhani and Miller, 2022). Randomization ensures that potential individual-level biases —such as variations in personality traits or cultural norms affecting response styles—are evenly distributed across each country sample rather than concentrated within specific subgroups of the samples (Barrera et al., 2024). In other words, while some participants over- or underreport their happiness, this should not systematically vary between groups, meaning it would not compromise the validity of the comparisons made in the analysis.

Several rationales guided the selection of the happiness question as the sole measure of happiness in this study. Firstly, this question adeptly captures the overall happiness of individuals without focusing on specific dimensions, ensuring a comprehensive understanding of participants' subjective wellbeing (Forgeard et al., 2011; Layard, 2009). Secondly, positioning this question as the first inquiry helps mitigate potential order effects on responses to subsequent questions. Finally, maintaining consistency with previous survey formats by including multiple wellbeing dimensions prevents the perception of an unconventional or overly brief survey design, which might otherwise influence response patterns.

### 2.5 Analytical approach

Embracing the commonly shared assumption that answers to subjective wellbeing questions are cardinal (Ferrer-i-Carbonell and Frijters, 2004), an Ordinary Least Squares (OLS) estimator was employed to assess the impact of exposure to positive or negative information (compared to neutral information) regarding macro-labor market opportunities on happiness. Specifically, the following equation was estimated:


(1) $$\begin{array}{l} Happiness_{ic}=\;\propto +\;pos_{ic}\psi +neg_{ic}\beta +X_{ic}\delta +\varepsilon_{ic} \end{array}$$

In this equation, *Happiness* represents the self-reported happiness of individual *i* in context *c*; *pos* indicates whether individual *i* in context *c* was exposed to positive information; *neg* indicates whether individual *i* in context *c* was exposed to negative information. Both *pos* and *neg* are components of the categorical treatment variable, assuming a value of one if a student received the negative information treatment (represented by the dummy variable *neg*), two if a student received the neutral information treatment (represented by the dummy *contr*), and three if a student received the positive information treatment (represented by the dummy *pos*). The reference category in this equation is *contr* (i.e., control group, neutral information treatment). The vector *Xic* encompasses individual covariates of individual *i* in context *c* known to influence happiness –particularly in studies focusing on youth populations in developed economies (see Layard and De Neve, 2023; Dolan et al., 2008 for details). These covariates include age, gender (where male = 1 and female = 0), social background (father's educational group: elementary or secondary = 4, high school or undergraduate = 3, graduate = 2, and master or PhD = 1), and subjective health (very good = 1, good = 2, fair = 3, poor = 4). To be consistent with the literature that consistently finds a U-shaped relationship between age and subjective wellbeing (Blanchflower, 2021), age squared is also added in the model. Social background is operationalized according to the father's educational background, inspired by the Erikson-Goldthorpe-Portocarero framework (EGP; Erikson et al., 1979). Respondents were asked to identify the educational group their father belonged to. In my experiments, the variable consisted of four categories: (4) Elementary or Secondary, (3) High School or Undergraduate, (2) Graduate, and (1) Master or PhD. Participants had to select one of the four categories. The error term ε_*ic*_ is included in the equation.

It is important to recognize that while the set of covariates mentioned above are widely applied in the economics of happiness research, their inclusion in a randomized experimental design is not strictly necessary for unbiased treatment effect estimates (Angrist and Pischke, 2009). However, including them is justified for three reasons. First, the relatively small and specific samples (students in the U.S. and Spain) mean that, while balance tests confirm the validity of the identification strategy, randomization may not perfectly balance covariates. Controlling for these variables mitigates any residual imbalances. Second, including these covariates improves the precision of treatment effect estimates.

At the same time, this study carefully addresses concerns about overcontrol bias. Including control variables that do not necessarily precede (induced) perceptions of macro-labor market opportunities–i.e., intervening variables or colliders– or those that might act as mediators (e.g., optimism or financial expectations), could underestimate the overall effect of these perceptions on happiness levels (Rohrer, 2018). While this concern is more significant in cross-sectional survey data analyses (Bartram, 2021), this study adopts a conservative approach to minimize such risks.

## 3 Results

### 3.1 Main results

The mean happiness scores exhibit remarkable similarity between students in both samples, with slightly higher scores reported by students at the University of Barcelona compared to those at East Stroudsburg University (i.e., 7.33 and 7.12 on the 0–10 Likert-scale, respectively). For a detailed table and description of the distribution of happiness across the two contexts, please see Supplementary File C. Regarding covariates, participants in the first sample tend to have slightly higher social backgrounds compared to those in the second sample. Additionally, the majority reported good or very good health in both country cases. Gender distribution reveals a substantial male majority in the University of Barcelona sample (70.75%), with no significant gender differences observed in happiness levels. Conversely, the East Stroudsburg University sample presents a more gender-balanced distribution, yet men appear to report higher levels of happiness than women (means of 7.43 and 6.88, respectively; statistically significant at the 5% level). Additional descriptive analyses of the variables included in the model (e.g., variance–distribution) as well as balance tests based on the pre-treatment characteristics of the participants are provided in Supplementary File C.^2^

To examine whether covariates are balanced across treatment groups, balance tests are presented in Tables 1, 2 based on pre-treatment characteristics.

Tables 1, 2 display the mean statistics of the covariates across treatment groups in the University Barcelona and East Stroudsburg University case studies, respectively. Both tables show that, in general, all groups are similar on the observed characteristics, so the identification strategy is valid. There are no statistically significant differences across treatment groups' covariates (i.e., students' age, gender, social background or subjective health) in the University of Barcelona case study. In East Stroudsburg University neither, except in the case of subjective health when comparing the neutral information group with the positive information group, at 10 percent significance level (i.e., 0.0926).

Experimental results are presented in Table 3. Column 1 displays the results for the University of Barcelona sample, whereas column 2 corresponds to the results obtained for the East Stroudsburg University. These columns illustrate how exposure to positive or negative information framing on macro-labor market opportunities predicts participants' happiness.

**Table 3:** Information-framing effects on happiness per context.

	**University of Barcelona (Spain) Happiness**	**East Stroudsburg University (U.S.) Happiness**
**Variables**	**(1)**	**(2)**
Positive information	0.562^**^	−0.427
	(0.259)	(0.306)
Negative information	−0.0104	−0.818^**^
	(0.290)	(0.321)
Age	−0.101	0.00376
	(0.302)	(0.178)
Age Sq.	0.00153	0.000158
	(0.00537)	(0.00253)
**Father Edu. (Ref: Master-PhD)**		
Graduate	0.197	−0.161
	(0.503)	(0.358)
High school	0.301	−0.907^***^
	(0.479)	(0.290)
Elementary	0.346	−0.500^*^
	(0.478)	(0.284)
Gender (male = 1 and female = 0)	0.0880	0.483^*^
	(0.242)	(0.261)
Subjective Health (1–4; very good to poor)	−0.486^**^	−0.751^***^
	(0.174)	(0.194)
Constant	9.135^*^	9.132^***^
	(4.130)	(2.616)
Observations	147	172
R-squared	0.130	0.172

Robust standard errors in parentheses.

^*^p < 0.1.

^**^p < 0.05.

^***^p < 0.01.

The findings reveal that participants' perceptions of macro-labor market opportunities significantly impacted their happiness in both contexts. Importantly, statistical significance and effect sizes appear to vary based on the type of treatment and the contextual setting. In the University of Barcelona sample, a strong effect of perceptions of macro-labor market opportunities on happiness is observed only when individuals are exposed to positive information (i.e., the change in happiness due to induced positive information is 0.562 on a 0–10 scale). This coefficient remains statistically significant at the 5 percent level (p=0.032) after accounting for individual covariates. Conversely, the induction of negative perceptions yields neither statistically significance (*p* = 0.972) nor strong effect sizes (−0.0104).

In the East Stroudsburg University sample, exposure to negative information results in a substantial decrease in reported happiness, with an effect size of nearly one point on the Likert scale (−0.818 on a 0–10 scale). This effect is statistically significant at the 5% level (*p* = 0.012), even after controlling for individual covariates. In contrast, exposure to positive information is associated with a smaller effect size of 0.427, which does not reach statistical significance (*p* = 0.166).

### 3.2 Heterogeneity

Tables 4, 5 examine the heterogeneous effects of the treatments based on social background and subjective health. While heterogeneity by gender was also explored, it did not yield any statistically significant results (see Table A1 in the Appendix).

**Table 4:** Heterogeneity by social background.

	**University of Barcelona (Spain) Happiness**	**University of Barcelona (Spain) Happiness**	**East Stroudsburg University (U.S.) Happiness**	**East Stroudsburg University (U.S.) Happiness**
**Variables**	**(1)**	**(2)**	**(3)**	**(4)**
Positive information	2.356^***^	0.560^**^	−0.548	−0.441
	(0.711)	(0.265)	(0.364)	(0.308)
Negative information	−0.00791	−0.858	−0.842^**^	−1.394^***^
	(0.291)	(0.913)	(0.327)	(0.374)
**Father Edu. (Ref: Master-PhD)**				
Graduate	0.636	−0.139	−0.0783	−0.825
	(0.539)	(0.554)	(0.436)	(0.532)
High school	0.705	−0.118	−1.071^***^	−0.961^***^
	(0.473)	(0.535)	(0.318)	(0.358)
Elementary	0.957^**^	0.124	−0.405	−0.889^**^
	(0.471)	(0.526)	(0.323)	(0.372)
**Father Edu. (Ref: Master-PhD)**				
**Postitive Info#Graduate**	−1.790^**^		−0.180	
	(0.809)		(0.700)	
**Postitive Info#High School**	−1.657^**^		0.467	
	(0.804)		(0.532)	
**Postitive Info#Elementary**	−2.255^***^		−0.212	
	(0.798)		(0.519)	
**Father Edu. (Ref: Master-PhD)**				
**Negative Info#Graduate**		0.914		1.614^**^
		(1.040)		(0.683)
**Negative Info#High School**		1.151		−0.338
		(0.999)		(0.614)
**Negative Info#Elementary**		0.498		0.947
		(1.041)		(0.628)
Age	−0.1000	−0.0387	0.00682	−0.0350
	(0.307)	(0.286)	(0.176)	(0.172)
Age Sq.	0.00157	0.000461	8.35e-05	0.000639
	(0.00542)	(0.00505)	(0.00250)	(0.00245)
Gender (male = 1 and female = 0)	−0.0299	−0.0974	0.480^*^	0.438^*^
	(0.243)	(0.248)	(0.262)	(0.257)
Subjective Health (1–4; very good to poor)	−0.485^***^	−0.464^***^	−0.720^***^	−0.782^***^
	(0.178)	(0.171)	(0.199)	(0.194)
Constant	8.794^**^	8.763^**^	9.067^***^	10.06^***^
	(3.979)	(3.750)	(2.608)	(2.656)
Observations	147	147	172	172
R-squared	0.155	0.142	0.179	0.209

Robust standard errors in parentheses.

^*^ *p* < 0.1.

^**^p < 0.05.

^***^p < 0.01.

**Table 5:** Heterogeneity by subjective health.

	**University of Barcelona (Spain) Happiness**	**University of Barcelona (Spain) Happiness**	**East Stroudsburg University (U.S.) Happiness**	**East Stroudsburg University (U.S.) Happiness**
**Variables**	**(1)**	**(2)**	**(3)**	**(4)**
Positive information	−0.520	0.610^**^	−0.451	−0.436
	(0.681)	(0.263)	(0.729)	(0.309)
Negative Information	−0.0246	1.013	−0.818^**^	−0.549
	(0.289)	(0.696)	(0.323)	(0.829)
Subjective Health (1–4; very good to poor)	−0.657^***^	−0.263	−0.757^***^	−0.713^***^
	(0.205)	(0.195)	(0.266)	(0.223)
**Postitive Info# Sub.Health**	0.587^*^		0.0134	
	(0.325)		(0.383)	
**Negative Info# Sub.Health**		−0.532^*^		−0.155
		(0.315)		(0.455)
**Father Edu. (Ref: Master-PhD)**				
Graduate	0.0411	0.0872	−0.161	−0.124
	(0.505)	(0.494)	(0.360)	(0.369)
High School	0.185	0.219	−0.906^***^	−0.879^***^
	(0.476)	(0.460)	(0.294)	(0.302)
Elementary	0.323	0.271	−0.500^*^	−0.470
	(0.478)	(0.459)	(0.285)	(0.298)
Age	−0.143	−0.0537	0.00343	−0.00199
	(0.299)	(0.299)	(0.176)	(0.178)
Age Sq.	0.00225	0.000569	0.000165	0.000296
	(0.00527)	(0.00531)	(0.00250)	(0.00253)
Gender (male = 1 and female = 0)	−0.0959	−0.0438	0.483^*^	0.476^*^
	(0.241)	(0.240)	(0.262)	(0.265)
Constant	10.32^**^	8.360^**^	9.144^***^	9.100^***^
	(3.954)	(3.935)	(2.555)	(2.669)
Observations	147	147	172	172
R-squared	0.150	0.152	0.172	0.172

Robust standard errors in parentheses.

^*^p < 0.1.

^**^p < 0.05.

^***^p < 0.01.

Regarding social background in the University of Barcelona sample, the findings indicate that the positive impact of particiants' perceptions of macro-labor market opportunities on happiness, when exposed to positive information, is particularly beneficial for those from higher social backgrounds (i.e., individuals whose father has a Master's or PhD degree) compared to the other groups. In particular, there is a constrast with those that come from low social backgrounds (i.e., whose father's highest education level is elementary school) who experience a strong decrease in happiness of 2.255 points on the 0–10 Likert scale scale, statistically significant at the 1% level.

In the East Stroudsburg University sample, the statistically significant negative impact of exposure to negative information on happiness appears to be partially mitigated for individuals from upper-middle backgrounds (i.e., those whose father attained graduate-level education) compared to participants from higher social backgrounds. This interaction effect, amounting to 1.614 points on the Likert scale, is statistically significant at 5 percent level.

Table 5 also reveals heterogeneous treatment effects based on subjective health, but only for the University of Barcelona sample. Interestingly, the results suggest that as individuals report poorer health conditions, they seem to be more sensitive to information exposure. Specifically, exposure to positive information leads to a 0.587 point increase in happiness, while exposure to negative information results in a comparable decrease of 0.532 points. Section 4.2 discusses these findings in greater detail.

## 4 Discussion

### 4.1 Main findings

This study reveals several key findings regarding its objective that constitute two main contributions. First, depending on the information treatment provided, perceptions of macro-labor market opportunities emerge as a strong determinant of individuals' happiness beyond individual conditions. This finding shows that perceptions of macro-labor market opportunities and happiness are strongly correlated. Thus, this study challenges the prevailing assumption in public policy and academic literature that the mere availability or improvement of labor market opportunities directly translates into individual's wellbeing (Chung and Mau, 2014). By providing empirical evidence of the importance of perceived macro-labor market opportunities, this study advances research on subjective wellbeing and offers valuable insights for wellbeing theory building and public policy. Policies aimed at improving labor market opportunities may be more effective if they also address how individuals perceive these opportunities. Ignoring perceptions risks producing a partial and incomplete understanding of labor market effects on wellbeing.

Second, the study identifies causal pathways linking perceptions of macro-labor market opportunities to happiness, with the effects depending on whether perceptions are positive or negative and on the broader socio-economic context. This is a particularly relevant contribution to happiness research, where causal evidence remains scarce. By employing a cross-country information provision natural field experiment, this study contributes to the growing research agenda on causality in subjective wellbeing. Additionally, it strengthens the emerging field of experimental sociology (Gereke and Gërxhani, 2019) by offering experimental evidence on the interplay between individual perceptions and societal conditions.

Furthermore, this study adds to the literature on information provision experiments, which often reveal substantial effects when examining subjective beliefs and perceived constraints (Haaland et al., 2023). In particular, it aligns with research on how individuals form expectations about the broader economy (Coibion et al., 2020; Roth and Wohlfart, 2020) and complements those that used natural field experiments (e.g., studies exploring voting behavior (Gerber et al., 2020; Cruz et al., 2024) or political activists (Hager et al., 2021; Cantoni et al., 2019).

Importantly, this study highlights that the observed causal effects are context-dependent. The finding that subtle variations in how macro-labor market opportunities are framed—introduced experimentally using truthful information—led to divergent impacts on individuals' happiness, even after controlling for individual characteristics, underscores the powerful role of perceptions in different contexts. These results suggest the presence of underlying psycho-social mechanisms that shape how individuals internalize information on the labor market and economic reality. The next section discusses these mechanisms in greater depth.

### 4.2 Cultural beliefs

Although this study's design and sample do not allow for an empirical identification of the mechanisms underlying the observed causal pathways, one plausible explanation is the influence of cultural cognitive biases. Affect valuation theory provides insights into how these biases might shape the relationship between perceptions of macro-labor market opportunities and happiness. Social psychology literature posits that individuals within the same culture often share specific cognitive tendencies, as cultural practices and meanings are deeply embedded and largely unquestioned (Kitayama and Markus, 2000). Culture, defined as “the collective mental programming of the human mind which distinguishes one group of people from another” (Hofstede, 1991, p. 5), plays a crucial role in shaping psychological processes (Di Maggio, 1994). Individuals internalize cultural norms from an early age, influencing how they perceive and process information, leading to cognitive biases that shape subjective wellbeing (Yiend et al., 2019).

Affect valuation theory suggests that individuals' perceptions of reality are influenced by discrepancies between their actual and ideal emotional states, with the ideal state serving as a culturally determined reference point (Tsai et al., 2006). Such discrepancies can trigger reference-point effects, impacting emotional wellbeing (Tversky and Kahneman, 1978).

Within this framework, the individualist-collectivist cultural dimension plays a center role and allows to understand how perceptions of macro-labor market opportunities can influence wellbeing (Becker et al., 2012; Diener and Suh, 2000; Kitayama et al., 1997). The University of Barcelona sample (Spain) represents a more collectivist cultural context, while the East Stroudsburg sample (U.S.) aligns with an individualist cultural framework (Hofstede et al., 2010). Research suggests that individuals in individualistic societies tend to exhibit optimistic cognitive biases, while those in collectivist societies lean toward more pessimistic biases (Chang and Asakawa, 2003; Sun et al., 2004; Rau et al., 2020). The experimentally induced positive or negative perceptions of macro-labor market opportunities may have affected students' happiness levels differently across cultural contexts, particularly when contradicting pre-existing beliefs.^3^

These findings align with theoretical and empirical research in cultural psychology (Bandura, 1999; Ivcevic and Kaufman, 2013; Jury et al., 2017). In an individualist society like the U.S., positive labor market information may have had little impact on happiness, as it aligns with prevailing individualist cultural ideals (e.g., the “Land of Opportunity”), whereas negative information may have disrupted expectations, decreasing happiness. In a more collectivist society like Spain, positive information may have improved happiness by challenging a culturally ingrained pessimism about labor market prospects, while negative information may have had a weaker effect, as it reinforced existing beliefs.

### 4.3 Heterogeneity discussion

This study highlights significant heterogeneous treatment effects based on social background and subjective health, both of which warrant further discussion. Regarding the University of Barcelona sample, findings indicate that the positive impact of participants' perceptions of macro-labor opportunities on happiness is particularly pronounced among individuals from higher social backgrounds, especially in comparison to those from lower social backgrounds. This aligns with previous research suggesting that individuals from privileged social origins tend to have greater social capital and more extensive professional networks, which enables them to respond more favorably to positive labor market information, as they can more effectively translate these signals into tangible opportunities (Bourdieu, 1986). In contrast, individuals from lower social backgrounds are generally less optimistic about future macroeconomic developments (Das et al., 2020). They are also more likely to perceive labor market outcomes as beyond their control (Manstead, 2018; García-Sierra, 2023). These psychological dynamics may have been particularly salient in the University of Barcelona sample due to the more pessimistic cultural biases discussed in the previous section, as well as the long-lasting psychological effects of past macroeconomic crises (see Giugni and Grasso, 2018), such as the 2008 Great Recession, which may have reinforced skepticism about the applicability of posititive labor market information to their career trajectories.

In the U.S. sample, a key finding is that the negative impact of exposure to negative macro–labor market information appears to be partially mitigated for individuals from upper–middle backgrounds (i.e., those whose fathers attained graduate-level education) compared to participants from higher social backgrounds. This could be explained by a confort–conditioning mechanism: whereas individuals from high social backgrounds may be less accustomed to confronting economic challenges, those from middle-high backgrounds–while still benefiting from economic security–may have more experience in coping with adversity, thereby developing greater resilience, and being less discourgaged by negative economic signals (Murphy and Allan, 2022). Furthermore, given the individualist cultural framework prevalent in the U.S., individuals from upper–middle social backgrounds (i.e., those with graduate-educated fathers) may have been raised with an emphasis on hard work, adaptability, and perseverance in the face of labor market uncertainties and life adveristies (Lareau, 2011). As a result, students from graduate-educated fathers may be better psychologically equipped to handle uncertainty, perceiving it as a natural part of career progression rather than a source of distress.

With respect to subjective health, findings from the University of Barcelona sample suggest that individuals in poored health exhibit heightened sensitivity to both positive and negative information about macro-labor market conditions. On one hand, exposure to positive information appears to serve as a buffer, helping individuals cope emotionally with their situation. On the other hand, negative information exarcebates their distress, reinforcing their sense of vulnerability. This latter result is consistent with vulnerability-stress models (Ingram and Luxton, 2005), which posit that individuals with greater physical, psychological, or social vulnerabilities are more likely to experience heightened emotional distress when exposed to stressors. More broadly, both findings on subjective health align with existing inequality literature suggesting that individuals facing adverse socioeconomic conditions tend to experience greater declines in subjective wellbeing when they also suffer from poor health (Wilkinson and Pickett, 2018).

### 4.4 Implications, caveats, and future directions

This study has several important caveats that point to potential avenues for future research. First, questions remain as to whether the differences observed between the samples can be fully attributed to cultural factors or if other contextual influences are at play. Given the constrains of the research design, the cultural cognitive bias argument remains speculative and should be interpreted with caution.

Second, while both samples represent WEIRD populations and were collected under stable macroeconomic conditions, I acknowledge that Barcelona and East Stroudsburg differ in several structural and sociocultural aspects, including urbanization levels, linguistic diversity, and regional economic structures. These factors may shape how individuals perceive labor market opportunities and wellbeing, and future research could further examine how these contextual dimensions influence the observed effects. Future studies could benefit from replicating these experiments in non-WEIRD settings or in societies characterized by stronger individualist or collectivist values (e.g., South-East Asian contexts such as South Korea or Japan) to assess the broader generalizability of these findings.

Third, concerns about the external validity of using undergraduate students as study participants warrant consideration. However, existing evidence supports the appropriateness of this population for examining social behavior and preferences (Exadaktylos et al., 2013; Snowberg and Yariv, 2021). Notably, the significant effects observed here, even with subtle framing manipulations based on macroeconomic information, suggest that stronger results may be anticipated in broader samples, especially given students' frequent exposure to economic data and the heightened attentiveness associated with their examination context.

More broadly, this study prioritizes internal validity to establish causal effects, addressing a key limitation of prior related correlational studies that, while valuable, cannot fully explore causal paths. Given the strong associations consistently found in observational research, this study seeks to complement that body of work by providing experimental evidence on the causal impact of perceptions of macro-labor market opportunities on subjective wellbeing. Future research should build on these findings by testing them in more diverse populations and real-world settings to enhance external validity.

Fourth, while the study employed an evaluative measure of wellbeing (*overall happiness*), which aligns with widely used subjective wellbeing assessments, an important limitation is the absence of purely affective measures. The design constraints of the experiment—particularly the need to preserve its natural setting—did not allow for the inclusion of real-time affective assessments. However, stronger or more immediate emotional responses may have emerged if the study had employed momentary affect measures, such as “*How happy are you right now?*” as used in Experience Sampling Methods (ESM) (Layard and De Neve, 2023; Diener et al., 2018). Future research could explore these real-time measures to better capture short-term emotional reactions to macroeconomic information. Moreover, it is crucial to acknowledge that both evaluative and affective assessments reflect individuals' self-reported perceptions of their happiness rather than objective measures of actual happiness. While self-reported measures have been shown to be reliable proxies for individuals' inner states (Pavot et al., 1991; Ekman et al., 1990; Sutton and Davidson, 1997), they do not constitute direct indicators of actual happiness. More objective approaches, such as behavioral or physiological markers (e.g., facial expressions, neural activity), could offer complementary insights into the underlying emotional responses.

Overall, this study enriches the literature on the labor market and subjective wellbeing by showcasing the way in which perceptions of macro-level labor market opportunities impact happiness. Depending on how information on these opportunities is framed and the context, emotional reactions may emerge, potentially influencing individual behavior and policy preferences, as proposed in the introduction.

The findings align with prior research on social mobility perceptions, political preferences, and wellbeing, underscoring that individuals in individualist societies often attribute personal outcomes and inequalities to individual effort rather than social factors, which correlates with reduced support for governmental intervention, labor market regulation, and redistributive policies (Alesina et al., 2018, 2004; Graham and Felton, 2006). As such, this study provides insights with implications for policy and organizational strategy, advancing our understanding of the psychological dynamics shaping labor market perceptions at the societal level. Recognizing these dynamics can guide strategies to address structural unemployment and inform the design of labor market policies in specific contexts. For instance, in the Spanish-Catalan setting, where the study was conducted, promoting entrepreneurship and economic activity by sharing positively framed but truthful macroeconomic data in public spaces–such as bus stops or subway stations–could help to foster optimism.

While the scope of this study does not allow for a deeper investigation into the mechanisms underlying the heterogeneous treatment effects observed, the findings nonetheless offer valuable policy implications. Policymakers may consider the physcological dynamics identified in this research when designing interventions tailored to specific social and cultural contexts. For instance, in the U.S., individuals from upper-middle social backgrounds exhibit adaptive strengths in managing labor market adversities, whereas in Spain, individuals with poorer subjective health demonstrate heightened sensitivity to information exposure. Notably, the interaction effects related to subjective health are significant only in the Spanish sample. This suggests the need for further investigation into potential cultural differences in how subjective health shapes emotional responses to macro-labor market information provision. Exploring these cross-context variations could enhance our understanding of the psychological and structural factors that shape individuals' perceptions of labor market opportunities.

Overall, this work underscores the importance of evaluating economic and social progress by integrating individuals' subjective assessments, which can contribute to both personal and societal prosperity.

## Data Availability

The raw data and Supplementary Online Materials of the study are available in article/Supplementary material. Further inquiries can be directed to the corresponding author.

## Footnotes

^1^In both contexts' negative treatments, it is noteworthy that answer category (d) diverges from that in the positive and control treatments. Specifically, it adopts a negative frame, accentuating the economic crisis to underscore the salience of the negative information. Conversely, for the positive and control treatments, a non-negative framing is applied across all answer categories.

^2^Supplementary File C presents the following tables: Supplementary Table 1. Happiness; Supplementary Table 2. Social background; Supplementary Table 3. Subjective health; Supplementary Table 4. Descriptive statistics of other covariates.

^3^Individualist cultures are typically associated with two interrelated cognitive biases: self-enhancement and optimism (Kitayama et al., 1997). The self-enhancement bias refers to individuals selectively focusing on their own successes while disregarding both personal failures and others' achievements, fostering a stable positive self-image (Kulik et al., 1986). The optimism bias, on the other hand, involves overestimating the likelihood of positive outcomes (Sharot, 2011). Other cognitive biases prevalent in individualist societies include the self-other bias (the tendency to view oneself as superior to others) and the illusion of control bias (an exaggerated belief in one's ability to influence outcomes) (Kobayashi and Brown, 2003). These biases can lead individuals in individualist societies to overestimate their opportunities (Alesina et al., 2018) and perceive themselves as more successful compared to individuals in collectivist cultures (Sun et al., 2004). In contrast, collectivist cultures are often characterized by a tendency toward pessimism bias (Rau et al., 2020; Chang and Asakawa, 2003), which involves overestimating the likelihood of negative outcomes and perceiving life opportunities as persistently inadequate (Kitayama et al., 1997). This bias also leads to reduced feelings of pride and increased feelings of guilt compared to those in individualist cultures (Eid and Diener, 2001). Cultural psychology suggests that pessimism bias serves as an adaptive mechanism in collectivist societies, reinforcing individuals' psychological dependence on the group to navigate adverse conditions and avoid the emotional costs associated with group disappointment or potential exclusion (Triandis, 2000).

## Appendix

**Table A1:** Heterogeneity by gender.

	**University of Barcelona (Spain)**	**University of Barcelona (Spain)**	**East Stroudsburg University (U.S.)**	**East Stroudsburg University (U.S.)**
**Variables**	**Happiness (1)**	**Happiness (2)**	**Happiness (3)**	**Happiness (4)**
Positive information	0.551	0.574^**^	–0.684^*^	–0.426
	(0.374)	(0.259)	(0.390)	(0.308)
Negative information	–0.010	–0.591	–0.804^**^	–0.755^*^
	(0.292)	(0.443)	(0.322)	(0.434)
Gender (male=1 and female=0)	–0.094	–0.318	0.259	0.523^*^
	(0.320)	(0.256)	(0.308)	(0.306)
**Postitive Info#Gender**	0.016		0.609	
	(0.453)		(0.541)	
**Negative Info#Gender**		0.784		–0.136
		(0.518)		(0.577)
Age	–0.100	–0.081	0.023	0.009
	(0.304)	(0.306)	(0.173)	(0.175)
Age Sq.	0.002	0.001	–0.000	0.000
	(0.005)	(0.005)	(0.002)	(0.002)
Father Edu. (Ref: Master-PhD) Graduate	0.197	0.188	–0177	–0.166
	(0.505)	(0.496)	(0.367)	(0.359)
High school	0.300	0.278	–0.918^***^	–0.924^***^
	(0.481)	(0.472)	(0.288)	(0.298)
Elementary	0.344	0.333	–0.501^*^	–0.518^*^
	(0.487)	(0.476)	(0.286)	(0.296)
Subjective Health	–0.486^***^	–0497^^***^^	–0.757^^***^^	–0.755^***^
(1–4; very good to poor)	(0.176)	(0.176)	(0.195)	(0.196)
Constant	9.302^***^ (3.958)	9.281^***^ (3.998)	8.974^***^ (2.548)	9.072^***^ (2.571)
Observations	147	147	172	172
R-squared	0.130	0.143	0.178	0.172

Robust standard errors in parentheses.

^***^ *p* < 0.01, ^**^ *p* < 0.05, ^*^ *p* < 0.1.
