# Supplementary material for: How perceptions of labor market opportunities predict happiness: evidence from natural field experiments
Source: Front Sociol. 2025 Apr 24;10:1527125. doi: 10.3389/fsoc.2025.1527125 (PMC12061025; doi:10.3389/fsoc.2025.1527125)
Supplement: Supplementary file 1 [file Supplementary_file_1.pdf]

Supplementary Online Materials for

How Perceptions of Labor Market Opportunities Predict Happiness: Evidence from Natural Field Experiments

Content

1. **Complementary design issues (SOM File A)**
2. **Power analysis (SOM File B)**
3. **Additional descriptive analysis (SOM File C)**
4. **Exams and experimental instructions (SOM File D)**
5. **Subjective wellbeing questionnaire (SOM File E)**

1. Complementary design issues (SOM File A)

Barcelona and East Stroudsburg Economic Conditions

Given the distinct historical trajectories of structural unemployment and labor market regulations, it is pertinent to assess the macroeconomic situations of Barcelona (Catalonia, Spain) and East Stroudsburg (Pennsylvania, the U.S.) at the time of the experiments. This period, spanning from the conclusion of 2018 to the initial quarter of 2019, represents a decade subsequent to the 2008 Economic Crisis and precedes the advent of the COVID-19 pandemic, characterized by stability and relative prosperity.

Barcelona, nestled in the northeastern Spanish region of Catalonia, has historically been recognized as one of the most prosperous areas in Spain and Southern Europe in terms of occupational opportunities and economic development. During the last trimester of 2018, the unemployment rate in Catalonia stood at 10.9%, marginally lower than the national average of 13.7% (Statistical Institute of Catalonia, 2024a). Additionally, the inter-annual variation in GDP for Catalonia was 1.8%, slightly below the Spanish average of 2.3% (Statistical Institute of Catalonia, 2024b).

East Stroudsburg, located in the interior of Pennsylvania, U.S., is part of the 'Rust Belt' or 'Manufacturing Belt,' experiencing a gradual deindustrialization process since the 1980s, particularly intensifying from the early 2000s. Despite this, at the time of the experiment, Pennsylvania boasted an unemployment rate of 4.3%, marginally higher than the national average of 4% (Bureau of Labor Statistics, 2024). Furthermore, the GDP growth rate for Pennsylvania was 2.5%, slightly surpassing the national average of 2.2% (Bureau of Economic Activity, 2024).

While these macroeconomic indicators underscore certain differences, it is important to note that both contexts were marked by relative stability and prosperity during the period under examination. Moreover, significant variations exist at the individual level. Students in Pennsylvania often grapple with substantial financial burdens to pursue a bachelor's degree in a public university, coupled with expenses for private health insurance. In contrast, access to higher education and healthcare in Catalonia and Spain is nearly free. For instance, some students at East Stroudsburg University disclosed that, in addition to private health insurance, they typically face tuition fees ranging from $40,000 to $60,000 USD. Conversely, at the University of Barcelona, bachelor's degree tuition fees range between €400 and €4,000.

Implementing Partners

The implementing partner in the Barcelona case study was the Department of Economic History, Institutions, Politics and World Economy, Faculty of Economics and Business at the University of Barcelona. This department offers courses related to the Spanish and European labor markets. The teaching session, offered by me, was developed within the 'European Integration' course. The course focuses on the institutions and policies of the European Union but also pays especial attention to the European and Spanish labor market dynamics. The course also provides case studies of small and medium enterprises operating in the international and European internal market.

In the Pennsylvania case study, the implementing partner was the Political Science and Economics Department at the East Stroudsburg University of Pennsylvania. The department offers courses on U.S. economy, institutions, policies and politics. Specifically, students participating in my experiment were attending different courses related to the U.S. labor market, international trade and European Union's transatlantic relations.

Framing of information treatments

In order to ensure the use of similar framing, but sufficiently adapted to the specific contexts, two points were addressed. First, it was necessary to use written language expressions that are common in Spanish and English. Using the exact same words and order in both languages would have otherwise made me lose control, because some students could have perceived some expressions being unusual in their language and thus bias the results. Second, it was necessary to include different words to adapt and describe the truthful macroeconomic historical context of each setting. For instance, the U.S. witnessed a decrease of the industrial sector and Spain a substantial increase in its public and private debt to invest in the real estate market.

References

Bureau of Economic Activity. (2024). Gross Domestic Product by State, First Quarter 2019. (Accessed, April 18). Washington, DC: (https://apps.bea.gov/regional/histdata/releases/0719gdpstate/index.cfm)

Bureau of Labor Statistics. (2024). *2019*. (Accessed, April 18). Washington, DC: (https://www.bls.gov/opub/mlr/2019/)

Statistical Institute of Catalonia. (2024a). *Labour, 2019*. (Accessed, April 18). Barcelona: Generalitat de Catalunya. (https://www.idescat.cat/indicadors/?id=conj&n=10218&lang=en&tema=treba&t=201804)

Statistical Institute of Catalonia. (2024b). *Main aggregates*. (Accessed, April 18). Barcelona: Generalitat de Catalunya. (https://www.idescat.cat/tema/macro?lang=en)

2. Power Analysis (SOM File B)

Based on the work of Wiswall and Zafar (2015) and subjective wellbeing studies, I expected a happiness treatment effect of around 1,5 on a 10 point Likert-scale. Assuming a standard deviation of 3 for the measurement, I needed a sample size of 126 subjects to achieve statistically significant results at 5% with a probability of 80%, or a sample size of 144 subjects with a probability of 85%. The final sample consists of 147 individuals in the University of Barcelona and 172 in the East Stroudsburg University.

S1 Fig. Power Calculations

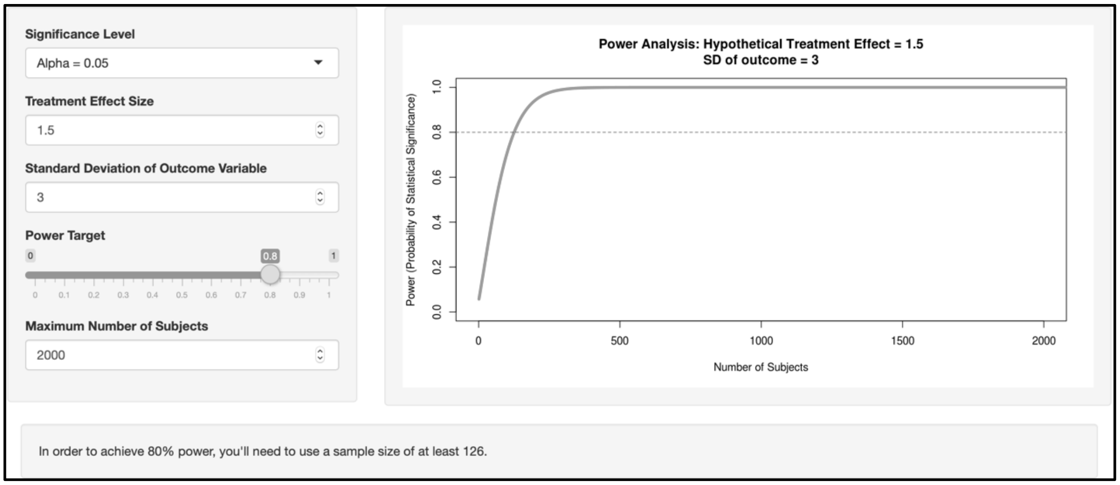

(source: https://egap.shinyapps.io/Power_Calculator/)

3. Additional descriptive analysis (SOM File C)

The following tables present a comparative overview of the descriptive statistics of the experimental subject pools. I start with the description of the dependent variable: subjective wellbeing. Subsequently, I present the other covariates of interest.

Table S1. Happiness

	University of Barcelona			East Stroudsburg University		
SWB	Freq.	Percent	Cum.	Freq.	Percent	Cum.
2				4	2.33	2.33
3	1	0.68	0.68	3	1.74	4.07
4	6	4.08	4.76	5	2.91	6.98
5	5	3.40	8.16	15	8.72	15.70
6	21	14.29	22.45	29	16.86	32.56
7	44	29.93	52.38	38	22.09	54.65
8	43	29.25	81.63	48	27.91	82.56
9	22	14.97	96.60	10	5.81	88.37
10	5	3.40	100.00	20	11.63	100.00
Total	147	1000		172	100.00	
Mean 7.333333				Mean 7.127907		
Std. Dev. 1.361573				Std. Dev. 1.788832		

In case of the University of Barcelona students, the mean value of their happiness is one unit higher (i.e., 7.333) than the value reported for Spanish people in general in the United Nations World Happiness Report 2018 (i.e., 6.310; 75). There are a number of reasons that could explain this difference. First, my sample consists mainly of higher education individuals, who usually report higher levels of happiness than the rest of the population (e.g. see: Dolan et al. 2008). Second, the experimental subjects live in Catalonia region, which has always been one of the most economically and socially developed regions in Spain. Based on a panel data study of Catalonia, Fernandez-Urbano and Kulic (2020) show that the levels of happiness in Catalonia are generally higher than in the rest of Spain.

In contrast, the mean value of East Stroudsburg University students' happiness is only slightly higher than the one mentioned in UN happiness report (i.e., 7.127 versus 6.886). According to the UN report, Finland is the happiest country in the world in 2018 (i.e., 7.632) whereas Burundi is the unhappiest (i.e., 2.905). A key observation here is that East Stroudsburg University students show more extreme values in terms of happiness than the University of Barcelona ones. For instance, whereas only 3.4% report a happiness level of 10 in the University Barcelona case, in East Stroudsburg University 11.63% of respondents report this level. Also, whereas the cumulative percentage of

individuals who report a happiness level of 3 or lower is 0.68% in the University of Barcelona case, in East Stroudsburg University it is 4.07%.

Table S2. Social background

	**University of Barcelona**			**East Stroudsburg University**		
	Freq.	Percent	Cum	Freq.	Percent	Cum
Father's educational background						
1 Master or PhD	8	5.44	5.44	5	2.91	2.91
2 Graduate	46	31.29	36.73	29	16.86	19.77
3 High School	54	36.73	73.47	72	41.86	61.63
4 Elementary	39	26.53	100.00	66	38.37	100.0
Total	147	100.00		172	100.00	
Mean	2.843537			3.156977		
Std. Dev.	.8813181			.804816		

Social background is operationalized according to the father's educational background, inspired by the Erikson-Goldthorpe-Portocarero framework (EGP; Erola et al., 2016; Meraviglia et al., 2018). It asks respondents to identify the educational group their father belonged to. In my experiments, the variable consisted of four categories: (4) Elementary or Secondary, (3) High School or Undergraduate, (2) Graduate and (1) Master or PhD. The participants had to choose one of the four categories. In both contexts, the standard deviation is around 0.8 and the highest percentage category corresponds to students whose father reached high-school or undergraduate education (i.e. middle social background). It could be argued that the majority of students in both countries do not come from high social backgrounds, as their fathers reached either high school or elementary education. Nonetheless, the Barcelona sample has a slightly higher social background than the one in Pennsylvania: 36.73% of the sample in Barcelona reported fathers who reached Master-PhD or graduate education, while only 19.77% of the Pennsylvania sample reached this level.

Table S3. Subjective health

	University of Barcelona			East Stroudsburg University		
	Freq.	Percent	Cum.	Freq.	Percent.	Cum.
1 Very Good	50	34.01	34.01	64	37.21	37.21
2 Good	66	44.90	78.91	79	45.93	83.14
3 Fair	27	18.37	97.28	27	15.70	98.84
4 Poor	4	2.72	100.00	2	1.16	100.00
Total	147	100.00		172	100.00	
Mean	1.897959			1.80814		
Std. Dev.	.7915192			.7361533		

Regarding subjective health, students could answer four categories: (4) Poor, (3) Fair, (2) Good, and (1) Very Good. As it could be expected from a sample of young students, the majority declared to have a good or very good subjective health perception in both case studies (i.e. mean of 1.8).

Table S4. Descriptive statistics of other covariates

University of Barcelona

Variable	Obs	Mean	Std. Dev.	Min	Max
Age	147	21.35374	2.719294	19	40
Gender	147	1.292517	.4564737	1 (M)	2 (F)

East Stroudsburg University

Variable	Obs	Mean	Std. Dev.	Min	Max
Age	172	20.36628	3.364698	18	52
Gender	172	1.55814	.4980582	1 (M)	2 (F)

In terms of gender (1=male; 2=female), the University of Barcelona sample consists of 43 females and 104 males. Therefore, there is a clear majority of males in the sample (70.75%). In any case, the mean of males' subjective well-being appears to be similar to that of females (i.e., 7.35 and 7.27, respectively). In contrast, in East Stroudsburg University case study, 76 individuals identified as male (44%) and 96 individuals identified as female (55%). In East Stroudsburg University men appear to be, on average, 0.5 happier than women in the 10 Likert scale of happiness (i.e. means of 7.43 and 6.88 respectively). This difference is statistically significant at the 5% level.

4. Exams and experimental instructions (SOM File D)

Exams

This section shows the English translation of the original exam students at University of Barcelona had to take. It is followed by the exam taken by the East Stroudsburg University students (*italics in questions four and five of both exams indicate places where alternative information was included for the treatments*). Horizontal lines indicate page separations.

University of Barcelona Exam

Exam

1. The Treaty of Paris (1951) was key to understanding the European Union of today. Which was its main objective?

   a. Economic integration.
   b. Peace.
   c. Fiscal integration
   d. Controlling Germany.

2. With its own peculiarities, nowadays most countries of the European Union have consolidated welfare states. At what time were most of the regimes established and promoted in Western Europe?

   a. At the end of the 19th century and/or the beginning of the 20th century.
   b. During the interwar period.
   c. After World War II: 1950s & 1960s.
   d. During the 1980s.

3. The way in which welfare states operate has been changing in most EU countries since the 1980s. Which has been the theoretical objective of the 'Activation Turn' paradigm?

   a. The dismantling of the welfare states.
   b. The maintenance of the welfare states.
   c. The expansion of the welfare states.
   d. Nothing related to the functioning of the welfare states.

---

4. Active Labour Market Policies (ALMPs) have become one of the most important elements of European social policy. During the last decade, the main purpose of ALMPs has been to *increase employment opportunities across* European countries. In this sense, the European Commission has been promoting them through the so-called 'Open Method of Coordination.' Which was the first country to promote ALMPs? At what time was it?

a. Sweden during in the interwar period.
b. Denmark in the 1970s.
c. UK in the 1980s.
d. Switzerland in the 1950s

5. *Fortunately, employment rates in Spain have been firmly raising. Furthermore, after one decade, employment rates are supposed to reach very soon the same levels as in 2008*. At what moment did Spain begin to increase its private and public debt to invest in the real-estate market and start the process of deindustrialization that lead to the state of the economy of 2008?

a. At the end of the Franco Regime.
b. At the beginning of the 1980s.
c. At the beginning of the 2000s.
d. *During 2006 and 2007*."

***

East Stroudsburg University Exam

Exam

1. The Treaty of Paris (1951) was key to understanding the European Union of today. Which was its main objective?

a. Economic integration.
b. Peace.
c. Fiscal integration.
d. Controlling Germany.

2. With its own peculiarities, nowadays most countries of the European Union have consolidated welfare states. At what time were most of the regimes established and promoted in Western Europe?

a. At the end of the 19th century and/or the beginning of the 20th century.
b. During the interwar period.
c. After World War II: 1950s & 1960s.
d. During the 1980s.

3. The way in which welfare states operate has been changing in most EU countries since the 1980s. Which has been the theoretical objective of the 'Activation Turn' paradigm?

a. The dismantling of the welfare states.
b. The maintenance of the welfare states.
c. The expansion of the welfare states.
d. Nothing related to the functioning of the welfare states.

4. Active Labor Market Policies (ALMPs) have become one of the most important elements of European social policy. During the last decade, the main purpose of ALMPs has been to *increase employment opportunities across European countries*. In this sense, the European Commission has been promoting them through the so-called 'Open Method of Coordination'. Which was the first country to promote ALMPs? At what time was it?

a. Sweden during in the interwar period.
b. Denmark in the 1970s
c. UK in the 1980s.
d. Switzerland in the 1950s.

5. *Fortunately,* in the U.S. context*, employment rates have been rising steadily and have already reached the same levels as in 2008. This fast recovery has been characterized* by an increased tertiary sector and a decreased industrial sector. In which moment did the U.S. experience the most important process of deindustrialization?

a. During the 1960s
b. During the 1980s.
c. During the 2000s.
d. *During 2006 and 2007.*

***

Experimental Instructions

This section shows the English translation of the original instructions for the exam sessions in the Barcelona case study. The instructions were the same for the Pennsylvania case study. At the start of the exam session:

Welcome. You are about to start the exam. Please use a pen only and remain silent until I tell you that you can begin to do the exam.

(once all exams and subjective well-being questionnaires are distributed)

You have now 30 minutes to complete the exam. When you have finished, you can answer a subjective wellbeing questionnaire that takes less than five minutes. You are not allowed to leave the room until every student has finished in order to avoid noise and disturbance.

(once all students completed the exam and the subjective wellbeing questionnaire)

Thank you very much. Apart from doing the exam, today you were also part of an experiment. If you wish, I need your consent with a written authorization that I will give you now. I guarantee you that the exam continues to be fully evaluated for your grades. If you have any questions in relation to the experiment, I will be happy to answer them now or if you prefer by email.

5. Subjective wellbeing questionnaire (SOM File E)

(For University of Barcelona students, the questionnaire was in Spanish).

This is an anonymous questionnaire. Please answer the following questions. Your anonymity is guaranteed.

Overall Level of Subjective Wellbeing

Taking all things together, how happy would you say you are? Note that 0 is Extremely Unhappy and 10 is Extremely Happy.

0 1 2 3 4 5 6 7 8 9 10

Taking all things into consideration, what is your level of satisfaction with life in general? Note that 0 is very dissatisfied and 10 is very satisfied.

0 1 2 3 4 5 6 7 8 9 10

Specific Dimensions of Subjective Wellbeing

**Positive Emotion**	0= Never 10= Always
How often do you feel joyful?	0 1 2 3 4 5 6 7 8 9 10
How often do you feel positive?	0 1 2 3 4 5 6 7 8 9 10
To what extent do you feel contented?	0 1 2 3 4 5 6 7 8 9 10

**Engagement**	0= Never 10= Always
How often do you become absorbed in what you are doing?	0 1 2 3 4 5 6 7 8 9 10
To what extent do you feel excited and interested in things?	0 1 2 3 4 5 6 7 8 9 10
How often do you lose track of time while doing something you enjoy?	0 1 2 3 4 5 6 7 8 9 10

Relationship

To what extent do you receive help and support from others when you need it?	0= Never 10= Always
	0 1 2 3 4 5 6 7 8 9 10
To what extent have you been feeling loved?	0= Not at all 10=A lot
	0 1 2 3 4 5 6 7 8 9 10
How satisfied are you with your personal relationships?	0= Not at all 10= Completely
	0 1 2 3 4 5 6 7 8 9 10

**Meaning**	0= Never 10= Completely

To what extent do you lead a purposeful and meaningful life?	0 1 2 3 4 5 6 7 8 9 10
To what extent do you feel that what you do in your life is valuable and worthwhile?	0 1 2 3 4 5 6 7 8 9 10
To what extent do you generally feel you have a sense of direction in your life?	0 1 2 3 4 5 6 7 8 9 10
**Accomplishments**	0= Never 10= Always
How much of the time do you feel you are making progress towards accomplishing your goals?	0 1 2 3 4 5 6 7 8 9 10
How often do you achieve the important goals you have set for yourself?	0 1 2 3 4 5 6 7 8 9 10
How often are you able to handle your responsibilities?	0 1 2 3 4 5 6 7 8 9 10
How do you perceive the state of the labor market opportunities in your country/region?	0= Terrible 10= Excellent<br>0 1 2 3 4 5 6 7 8 9 10

***

Post-Survey Questions

Age:

Gender:

Father Educational Background: Elementary /Secondary/ Undergraduate/ Graduate

Health Perception: Very Poor /Poor /Fair /Good /Very Good
