# Supplementary material for: How perceptions of labor market opportunities predict happiness: evidence from natural field experiments
Source: Front Sociol. 2025 Apr 24;10:1527125. doi: 10.3389/fsoc.2025.1527125 (PMC12061025; doi:10.3389/fsoc.2025.1527125)
Supplement: Supplementary file 2 [file Supplementary_file_2.pdf]

```
*"How Perceptions of Labour Market Opportunities Predict Happiness: Evidence from Natural Field Experiments"

clear all
import excel "/Users/name/Desktop/RAW Data.xlsx", sheet("1") firstrow clear

***** Rename & Generate Varaibles *********************

rename OutcomeHappiness happy
rename SocialBackground background
rename SubjectiveHealth health
rename Age age
rename Invididual id
rename Treatment treat
rename Gender gender
replace gender =0 if gender==2

gen age2 = age*age

gen pos = (treat==3)
gen neg = (treat==1)
gen contr = (treat==2)

***** Descriptive analysis ***************************

preserve
keep if context==1
describe happy pos neg age background gender health
summarize happy pos neg age background gender health
tabulate happy
restore

preserve
keep if context==0
describe happy pos neg age background gender health
summarize happy pos neg age background gender health
tabulate happy
restore

*Balance Test
ssc install iebaltab
preserve
keep if context==1
iebaltab age gender backg* health, grpvar(treat) save(balance_Barcelona.xlsx) pttest
restore

preserve
keep if context==0
iebaltab age gender backg* health, grpvar(treat) save(
```

```
balance_EastStroudsburg.xlsx) pttest
restore

***** Main Analysis **************************************

*University of Barcelona
reg happy pos neg age age2 i.background gender health if context ==1, robust
outreg2  using "Table1.xls", se nocons bdec(3) title( "Information-framing effects on happiness per context.") see label

*East Stroudsburg University
reg happy pos neg age age2 i.background gender health if context ==0, robust
outreg2  using "Table1.xls", se nocons bdec(3) title( "Information-framing effects on happiness per context.") see label

*******Heterogeneity****************************************
*By gender
reg happy pos##i.gender neg age age2 i.background health if context==1, robust
outreg2  using "TableA1.xls", se nocons bdec(3) title( "Heterogeneity by Gender") see label
reg happy pos neg##i.gender age age2 i.background health if context==1, robust
outreg2  using "TableA1.xls", se nocons bdec(3) title( "Heterogeneity by Gender") see label
reg happy pos##i.gender neg age age2 i.background health if context==0, robust
outreg2  using "TableA1.xls", se nocons bdec(3) title( "Heterogeneity by Gender.") see label
reg happy pos neg##i.gender age age2 i.background health if context==0, robust
outreg2  using "TableA1.xls", se nocons bdec(3) title( "Heterogeneity by Gender") see label

*By social background
reg happy pos##i.background neg age age2 gender health if context ==1, robust
outreg2  using "Table3.1.xls", se nocons bdec(3) title( "Heterogeneity by Social Background") see label
reg happy neg##i.background pos age age2 gender health if context ==1, robust
outreg2  using "Table3.1.xls",se nocons bdec(3) title( "Heterogeneity by Social Background") see label
reg happy pos##i.background neg age age2 gender health if context ==0, robust
outreg2  using "Table3.1.xls",se nocons bdec(3) title( "Heterogeneity by Social Background") see label
reg happy neg##i.background pos age age2 gender health if context
```

```
"Heterogeneity by Social Background") see label

*By subjective health
reg happy pos##c.health neg age age2 i.background gender if context==1, robust
outreg2  using "Table3.2.xls", se nocons bdec(3) title("Heterogeneity by Subjective Health") see label,se nocons bdec(3) title("Heterogeneity by Subjective Health") see label
reg happy neg##c.health pos age age2 i.background gender if context==1, robust
outreg2  using "Table3.2.xls", se nocons bdec(3) title("Heterogeneity by Subjective Health") see label,se nocons bdec(3) title("Heterogeneity by Subjective Health") see label
reg happy pos##c.health neg age age2 i.background gender if context==0, robust
outreg2  using "Table3.2.xls", se nocons bdec(3) title("Heterogeneity by Subjective Health") see label,se nocons bdec(3) title("Heterogeneity by Subjective Health") see label
reg happy neg##c.health pos age age2 i.background gender if context==0, robust
outreg2  using "Table3.2.xls", se nocons bdec(3) title("Heterogeneity by Subjective Health") see label,se nocons bdec(3) title("Heterogeneity by Subjective Health") see label

```
